# A comprehensive mobile nutritional application is associated with improved time efficiency and user experience in managing hospitalized children with malnutrition

**DOI:** 10.1186/s12887-025-06423-9

**Published:** 2025-12-16

**Authors:** Settachote Maholarnkij, Chonnikant Visuthranukul, Eakkarin Mekangkul, Jaraspong Uaariyapanichkul, Sirinuch Chomtho

**Affiliations:** 1 Division of Nutrition, Department of Pediatrics, King Chulalongkorn Memorial Hospital, The Thai Red Cross Society, Bangkok, 10330 Thailand; 2 Center of Excellence in Pediatric Nutrition, Division of Nutrition, Department of Pediatrics, Faculty of Medicine, Chulalongkorn University, Bangkok, 10330 Thailand; 3 Division of Nutrition , Department of Pediatrics, Faculty of Medicine, Chulalongkorn University, Bangkok, 10330, Thailand

**Keywords:** Hospitalized children, Malnutrition, Mobile nutritional application, Nutritional management, Target energy and protein intakes

## Abstract

**Background:**

Despite the growing use of mobile applications in healthcare, few tools for children comprehensively support the entire nutritional care process. Novel technologies have been shown to encourage healthcare teams to initiate nutritional management and reduce the workload of nutrition support teams. This study aimed to assess the feasibility and initial impact of the iNutri application, compare its time efficiency for nutritional management with that of the conventional method, and assess user satisfaction and comfort.

**Methods:**

The iNutri application, a comprehensive mobile platform for integrating nutritional assessment, management, and monitoring, was developed. In a prospective pilot study, pediatric residents, as members of the nutritional care team, used iNutri as a tool within the conventional nutritional care process for hospitalized children with malnutrition. The time to achieve the target energy and protein intakes, and the length of hospital stay were assessed. A structured satisfaction survey with a 5-point Likert scale was used to assess user feedback. User feedback on time efficiency, satisfaction, and comfort was analyzed.

**Results:**

Sixty pediatric inpatients with malnutrition were included, with 14 achieving early nutrient requirements. Shorter hospital stays were observed in the early target achievement group (*p* = 0.003). Compared with the conventional method, the application was associated with a significant reduction in the time required for the nutritional care process by 16 min (*p* < 0.001). Positive feedback was received regarding the ease of using iNutri (4.4/5), the comprehensiveness of nutritional details (4.2/5), confidence in performing 24-hour dietary recall (4.1/5), initiating enteral or parenteral nutrition (4/5), and mastery in managing the nutritional care process (4/5).

**Conclusions:**

The iNutri application suggests the potential to improve the efficiency of nutritional care for pediatric inpatients with malnutrition, demonstrating an observed reduction in the time required for the nutritional care process. Positive feedback emphasized its ease of usability and effectiveness. Early achievement of target nutrient intake may be associated with shorter hospital stays. These findings are valuable for informing the design of future, large-scale randomized controlled trials.

**Trial registration:**

Thai Clinical Trials Registry TCTR20220319001. Registered 18 March 2022.

**Supplementary Information:**

The online version contains supplementary material available at 10.1186/s12887-025-06423-9.

## Introduction

Malnutrition is a major concern when caring for pediatric inpatients [1]. The prevalence of malnutrition among hospitalized children in Europe ranges from 6% to 30%, on the basis of various diagnostic criteria for malnutrition [2]. In Thailand, recent multicenter data reported a prevalence of 44.5% among hospitalized children, with acute and chronic malnutrition rates of 14.3% and 23.6%, respectively, highlighting a considerable burden in this setting [3]. The more severe the malnutrition is, the greater the risk of complications and unfavorable outcomes, such as infections, weight loss, and longer lengths of stay (LOS) [2, 4]. Inappropriate nutritional support, such as delayed enteral feeding, is one of the potential risks associated with malnutrition. During hospitalization, factors such as unnecessary feeding interruption, fluid restriction, major surgery, and severe illness can hinder the provision of adequate nutrition [5, 6]. To address these issues, scoring systems and screening tools were invented for to help pediatricians detect patients at risk of nutritional depletion [7–9]. Among those tools, the Screening Tool for Risk on Nutritional Status and Growth (STRONGkids) was developed and validated in multicenter hospitalized children as a notable nutritional screening tool [10]. However, despite the availability of such tools, the burden of malnutrition among hospitalized pediatric patients, particularly in developing countries, has remained significant [11], especially in Thailand [12]. 

Despite the importance of nutritional support, clinical nutrition has received little emphasis in traditional medical education, and most physicians are hesitant to prescribe enteral nutrition (EN) or parenteral nutrition (PN) [13]. To address this challenge, novel technologies in nutrition care have been developed, particularly for adult patients [14–16]. A retrospective study in the United States evaluated the benefits of EN application in facilitating the ordering of EN by internal medicine residents in a medical intensive care unit [17]. The proportion of patients who obtained EN and achieved energy targets increased after the introduction of the application. Another quasi-experimental study in Thailand compared the Nutrition Educational Prototype-based Smartphone Web Applications (NEPSAs) with standard hospital leaflets as nutrition-based educational tools for colorectal cancer patients [18]. The results revealed that NEPSAs improved patients’ appetite, nutritional parameters, and biochemical profiles. Another digital application, the ARIETI, was developed for calculating dietary intake in Brazil [19]. This instrument demonstrated high reliability and enabled nutritionists to detect nutritional risks associated with insufficient caloric intake 250 s faster.

Moreover, novel technologies have been invented for adult patients; however, nutritional applications for pediatric patients are scarce and most of them focus on obesity, self-monitoring, or educational purposes with a limited sample size in each study [16, 20–23]. Few nutritional applications for children have focused on the nutrition care process; for example, Nutrikids, developed for simplifying dietary assessment [24], and EatBaby, created for proper nutritional management in children with congenital heart disease undergoing surgery [25]. According to the aforementioned studies, there is still a research gap in the development of a comprehensive tool that streamlines the entire nutritional care process. In Thailand, the shortage of autonomous and professional clinical dietitians further increases the burden on pediatricians to manage nutritional care independently. Therefore, we developed a mobile nutritional application for performing nutritional care processes more effectively. The primary objective of this pilot study was to assess the feasibility and initial impact of the application during the nutrition care process by determining the number of patients who achieved target energy and protein intakes. The secondary objectives were to compare the time required for nutritional management via the application method with that via the conventional method and to assess the satisfaction and comfort of pediatric residents.

## Methods

### Application development phase

#### Application function

The iNutri mobile nutritional application was developed by Settachote Maholarnkij, Chonnikant Visuthranukul, and Jaraspong Uaariyapanichkul in collaboration with Pan Apace Co., Ltd. (Bangkok, Thailand), with funding support from the King Chulalongkorn Memorial Hospital (KCMH), Thailand, through the Digital-Based Innovation Grant (iNutri: Comprehensive Mobile Nutritional Application; Asset Code: 02–2566-7690-003-02–0003). The application was designed as a cross-platform mobile tool, with data securely stored on the KCMH Information Technology Department server. Access was limited to verified pediatric residents via a login system, and anonymized patient codes (separate from hospital numbers) were generated to maintain confidentiality. Furthermore, various functions were incorporated into the iNutri application, which included the following parts:

##### Growth database

The Thai standard growth chart was applied in this study. In Thailand, the World Health Organization (WHO) Child Growth Standards for children from 0 to 5 years old [26] were applied in the national standard growth chart, given the details of database collection. For children aged 6–19 years, the Thai standard growth chart is based on the database from the Department of Health, Ministry of Public Health, Thailand Growth Reference, which is considered more appropriate for the Thai context [27]. 

##### Nutritional risk screening tool

The STRONGkids screening tool [9] was included to identify malnutrition risk. For clarity, the questions and scoring system are summarized in Table 1.

**Table 1:** STRONGkids screening tool for determining nutritional status and growth risk

Item	Sub-questions	Score
1. Subjective clinical assessment	Is patient in a poor general nutritional state?	Yes = 1, No = 0
2. Risk for malnutrition	Is there an underlying illness with risk for malnutrition or expected major surgery?	Yes = 2, No = 0
3. Nutritional intake and losses	During the last few days, has the child experienced reduced food intake, excessive diarrhea and/or vomiting, pre-existing nutritional intervention, or an inability to eat adequately due to pain?	Yes = 1, No = 0
4. Weight loss or poor weight gain	Is there weight loss or no weight gain (infants < 1 year) during the last few weeks/months?	Yes = 1, No = 0
Total Score	Sum of all items	0–5

Risk Categories:

• 0 points: Low risk

• 1–3 points: Moderate risk

• 4–5 points: High risk

*STRONGkids* Screening Tool for Risk on Nutritional Status and Growth

##### Nutritional assessment

Nutritional status was classified according to the WHO [28]. Children with weight-for-length/height between − 2SD and − 3SD or below − 3SD were defined as having moderate or severe wasting, respectively. Similarly, a length/height-for-age between − 2SD and − 3SD or below − 3SD indicated moderate or severe stunting, respectively.

##### Dietary assessment

A 24-hour dietary recall was analyzed via the Institute of Nutrition, Mahidol University CALculation (INMUCAL) version 4.0 program [29]. The application allows the input of various dietary sources, including breast milk, formula milk, modular formula, ultrahigh-temperature (UHT) milk, blenderized diets, and EN formulas. Individualized and commercial PN options are also available.

##### Determination of target energy and protein intakes

For the EN, the targets were selected on the basis of the Thai Dietary Reference Intakes (DRI) 2020 [30]. For the PN, requirements followed the European Society for Paediatric Gastroenterology, Hepatology and Nutrition (ESPGHAN)/European Society for Clinical Nutrition and Metabolism (ESPEN)/European Society for Paediatric Research (ESPR)/Chinese Society for Parenteral and Enteral Nutrition (CSPEN) guidelines for pediatric PN [31, 32]. The individualized target energy and protein intakes were further adjusted on the basis of the patient’s current nutritional status by multiplying the EN DRI energy target by the degree of wasting severity.

##### Long-term monitoring

The secure storage of patient data on the hospital server facilitated longitudinal monitoring of growth trajectories and nutrient intake for each patient.

##### Archives of food exchange

A built-in database on food exchange and portion sizes was included to support pediatric residents in performing accurate 24-hour dietary recalls.

##### User feedback

User feedback was assessed via a newly developed structured satisfaction survey embedded at the end of the application. The survey included several items rated on a 5-point Likert scale (1 = least satisfied, 5 = most satisfied) to evaluate various aspects of the application. The full survey interface is provided in Supplemental Fig. 1.

#### Overall performance of the iNutri application

To summarize how the iNutri functions, users were required to complete the patient’s age, sex, and anthropometric measurements in the designated fields, along with completing the STRONGkids screening tool. The application was designed to directly streamline and enhance the conventional nutrition care process by automating several time-consuming steps. The workflow for pediatric residents using the application is as follows:

- Automated nutritional screening and assessment: Once the initial data are entered, the nutritional status and the malnutrition risk score of the patient are calculated automatically. This immediate, automated calculation is a key time-saver, eliminating the need for manual scoring and interpretation.
- Simplified dietary assessment: After that, users select sources of nutrients (both EN and PN) and enter dietary intake. The application’s built-in food exchange database aids in accurate dietary recall. Once the data are entered, the patient’s energy, carbohydrate, protein, and fat intakes are computed autonomously. This replaces the manual, and often error-prone, process of looking up nutrient values and calculating totals.
- Individualized target intake determination: The application then presents individualized suggestions for target energy and protein intake on the basis of the patient’s nutritional status.
- Longitudinal monitoring: The long-term monitoring module is shown only upon subsequent access with the same patient’s information. This feature allows for the longitudinal tracking of growth and nutrient intake trends without the need to re-enter all baseline information.

**Fig. 1:**
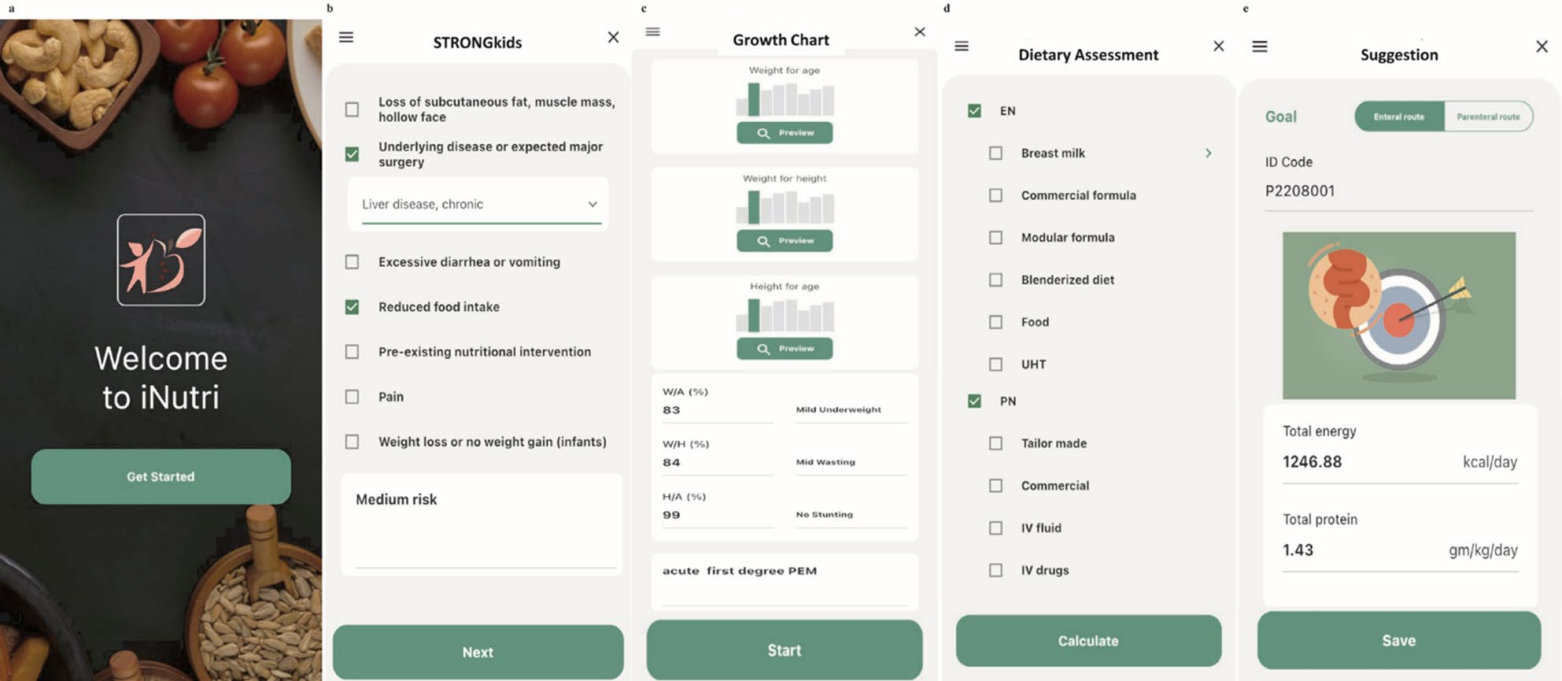
iNutri application screenshots. The five panels show the interface of the iNutri application with the user. Panels from left to right: (**a**) the initial screen; (**b**) the screen for assessment of risk of malnutrition; (**c**) the screen for interpretation of nutritional status; (**d**) the screen for the user to perform 24-hour dietary recall in both enteral and parenteral nutrition; (**e**) the final screen with suggestions for the target energy and protein intake

This application-assisted workflow directly contrasts with the conventional paper-based method, where each of these steps, from calculating nutritional scores to computing nutrient intakes and determining target requirements, must be performed manually. The automation provided by iNutri significantly reduces the time burden and potential for human error, which is why the data entry process was observed to take approximately 5–10 min. Screenshots of the iNutri application interface are displayed in Fig. 1.

#### Reliability test of the iNutri application

Prior to implementation, the iNutri application was pretested by general pediatricians and pediatrician nutrition specialists, and their feedback was incorporated to refine the interface and enhance usability. The comprehensive data entry process during pretesting required approximately 5–10 min, which was 10–15 min shorter than the estimated time using the conventional paper-based method, suggesting a potential reduction in the time burden for pediatric residents. The correlation between the standard method and iNutri application in the dietary assessment was examined. A paper-based 24-hour dietary recall was conducted by professional dietitians, whereas dietary assessments using the iNutri application were performed by general pediatricians. The target was to achieve an expected reliability, expressed as an intraclass correlation coefficient (ICC) of 0.90, with a precision of 5%, resulting in a required sample size of 65 participants [33]. 

### Clinical phase

#### Using the iNutri application for nutrition care

The study was designed as a single-arm prospective study conducted from August 2022 to June 2024. Written informed consent was obtained from parents and from pediatric residents. For children aged 7 years and older, assent was also obtained in accordance with ethical guidelines. This study was approved by the Institutional Review Board of the Faculty of Medicine, Chulalongkorn University, Bangkok, Thailand (IRB No.328/64).

##### Participants

The inclusion criteria were pediatric patients admitted to the pediatric intensive care unit (PICU) or general ward at KCMH, aged between 1 month and 18 years, with malnutrition [28] or a medium or high risk of malnutrition [9], with LOS of at least 5 days, who were approved by their parents and who volunteered to participate in the study. The exclusion criteria included growth variations from genetic factors or skeletal disorders affecting length/height. The data of demographics, principal diagnosis, underlying disease, anthropometric measurements, nutritional status, STRONGkids score, history of surgery, consultation with the pediatric nutrition unit, signs of nutrient deficiency, fluid restriction, feeding intolerance, energy and protein intake, target energy/protein intake, time to reach target, and LOS were collected. The patient screening and enrollment process is detailed in the flowchart provided in Supplemental Fig. 2.

##### Training and orientation of residents

All pediatric residents were instructed on the use of the iNutri application prior to participation. The research team provided a detailed step-by-step instructional video, which residents could view multiple times and select specific sections as needed. Additionally, a live hands-on training session was conducted by the research team (physicians), during which residents received guidance and could ask questions in detail.

##### Clinical use of the iNutri

During the study, residents who agreed to participate entered each patient’s age, sex, anthropometric measurements, and dietary recall into the application during two sessions: within 24 h of admission and upon reaching at least 80% of target energy and protein intakes. Application administrators were available to assist residents during these sessions. Patients typically achieve target energy/protein intake within approximately 7 days (168 h), and achievement was defined as ≥ 80% of the calculated targets. Feeding intolerance was defined as recurrent gastric residual volume > 50% of the administered volume, gastric distension, vomiting, or diarrhea [34]. Finally, the patients were categorized into early and late achievement groups on the basis of the time required to reach at least 80% of their individualized target energy and protein intakes. Early achievement was defined as reaching the target within 7 days of admission, whereas late achievement was defined as reaching the target after 7 days. Factors associated with early achievement were analyzed.

#### Satisfaction and comfort survey

Informed consent was obtained from all the pediatric residents. This study was approved by the Institutional Review Board of the Faculty of Medicine, Chulalongkorn University, Bangkok, Thailand (IRB No.328/64). To compare the time required for nutritional management, the duration of the nutritional care process performed by residents was recorded during the standard paper-based method (before the introduction of the iNutri application) and the application-assisted method (after introduction). In the inpatient setting, the nutritional care process was routinely initiated and performed by pediatric residents. The recorded duration referred exclusively to the residents’ process, both with and without the iNutri application. Pediatric nutrition fellows were consulted afterward if needed, but this was not included in the time measurement. The satisfaction and comfort of the pediatric residents who used the application were also assessed.

### Statistical analysis

Continuous variables are presented as the means and standard deviations (SDs) for normally distributed data or as medians (Q1, Q3) for nonnormally distributed data. Categorical variables are presented as frequencies and percentages. To assess statistically significant differences between independent groups, Student’s t-test was used for continuous variables with a normal distribution, whereas the Mann-Whitney U test was used for nonnormally distributed data. For categorical variables, a Pearson Chi-square test or Fisher’s exact test was used as appropriate. For paired samples, Student’s t-test or Wilcoxon signed-rank test was used for continuous variables, depending on the distribution of the data. To identify factors associated with early achievement of target energy and protein intake, a multiple logistic regression model was used. We initially performed a univariate analysis with a comprehensive set of variables from the baseline patient characteristics (Table 2), which included malnutrition severity (e.g., wasting, stunting), main diagnosis, admission location (PICU vs. general ward), and all individual items from the STRONGkids tool. All variables with a p-value of less than 0.05 in the univariate analysis were then included in the final model to control for potential confounders. Akaike’s information criterion (AIC) and area under the receiver operating characteristic curve (auROC) were used for the model selection criteria. A statistically significant difference was considered when the *p*-value was < 0.05. All analyses were conducted via STATA version 16.1 (STATA Statistical Software: Release 16. College Station, TX: STATA Corp LLC. 2019).

**Table 2:** Baseline demographic and clinical characteristics of patients classified by early and late achievement of target energy and protein intakes^†^ (*n* = 60)

	Early achievement^†^(*n* = 14)	Late achievement^†^ (*n* = 46)	Total (*n* = 60)	*p*-value
Age (years)	0.96	1.46	1.38	0.599
	(0.83, 6.5)	(0.58, 3.08)	(0.67, 4.71)	
Male	9 (64.3)	30 (65.2)	39 (65)	0.949
Female	5 (35.7)	16 (34.8)	21 (35)	
Admission				0.854
PICU	8 (57.1)	25 (54.3)	33 (55)	
General ward	6 (42.9)	21 (45.7)	27 (45)	
STRONGkids items^‡^				
1.	1 (7.1)	3 (6.5)	4 (66.7)	1.000
2.	12 (85.7)	38 (82.6)	50 (83.3)	1.000
3-1	3 (21.4)	3 (6.5)	6 (10)	0.133
3-2	12 (85.7)	38 (82.6)	50 (83.3)	1.000
3-3	2 (14.3)	21 (45.7)	23 (38.3)	0.035*
3-4	10 (71.4)	38 (82.6)	48 (80)	0.448
4.	0 (0)	6 (13)	6 (10)	0.320
Risk of malnutrition				1.000
Medium	13 (92.9)	43 (93.5)	56 (93.3)	
High	1 (7.1)	3 (6.5)	4 (6.7)	
Weight-for-age	-0.05	-1.48	-1.19	0.001*
z-score	(-0.72, 1.00)	(-2.67, -0.92)	(-2.18, -0.31)	
Weight-for-length/height	0.84	-1.06	-0.88	0.008*
z-score	(-1.09, 1.97)	(-2.33, -0.29)	(-2.05, 0.37)	
Length/height-for-age	-0.69	-1.28	-0.99	0.133
z-score	(-1.30, 0.01)	(-2.79, -0.12)	(-2.73, -0.12)	
Malnutrition	2 (14.3)	27 (58.7)	29 (48.3)	0.005*
Wasting				0.377
Moderate	2 (14.3)	9 (19.6)	11 (18.3)	
Severe	0 (0)	6 (13)	6 (10)	
Stunting				0.100
Moderate	0 (0)	7 (15.2)	7 (11.7)	
Severe	1 (7.1)	11 (23.9)	12 (20)	
Consultation	4 (28.6)	24 (52.2)	28 (46.7)	0.140
Signs of nutrient deficiency	1 (7.1)	7 (15.2)	8 (13.3)	0.667
Fluid restriction	3 (21.4)	13 (28.3)	16 (26.7)	0.740
Feeding intolerance	4 (28.6)	11 (23.9)	15 (25)	0.734
Vasopressor medication used	2 (14.3)	12 (26.1)	14 (23.3)	0.485
Sedative medication used	6 (42.9)	19 (41.3)	25 (41.7)	0.918
Obtained liver transplantation operation	0 (0)	3 (6.5)	3 (5)	1.000
Obtained bowel surgery	0 (0)	1 (2.2)	1 (1.7)	1.000
Time to achieve target energy intake (hours)	108.5 (41, 150)	394.0 (257, 798)	257.0 (150, 430)	< 0.001*
Change of weight-for-length/height	0.07	0.15	0.14	
z-score	(-0.70, 0.52)	(-0.16, 0.38)	(-0.18, 0.41)	0.447
Length of stay (days)	12.0 (8, 18)	24.0 (14, 41)	21.5 (13, 34)	0.003*
Death	0 (0)	3 (6.5)	3 (5)	1.000

Categorical variables are expressed as *n* (%) and continuous variables are expressed as median (Q1, Q3). Chi-square or Fisher’s exact tests were used for categorical data. Mann-Whitney U test was used for continuous data

* Significant differences between groups (*p* < 0.05)

^†^ Early achievement was defined as reaching the target within 7 days of admission, while late achievement was defined as reaching the target after 7 days

^‡^ *STRONGkids* Screening Tool for Risk on Nutritional Status and Growth

(1) Subjective clinical assessment (0 or 1 point), (2) High-risk disease (0 or 2 points), (3) Nutritional intake and losses-reduced intake in the past few days, diarrhea, vomiting, or pre-existing nutritional intervention (0 or 1 point), and (4) Weight loss or poor weight gain (0 or 1 point). The total score ranges from 0-5, classified as low (0), moderate (1-3), and high (4-5) risk of malnutrition

*PICU* pediatric intensive care unit

## Results

### Dietary assessment reliability

The reliability of carbohydrate, protein, fat, and energy intake estimates obtained from the iNutri application was excellent, with ICC values (95% CI) of 0.998 (0.997–0.999), 0.994 (0.990–0.996), 0.998 (0.997–0.999), and 0.999 (0.998–0.999), respectively, based on two-way random-effects models.

### Evaluation of factors associated with early achievement of target nutrient intake

A total of 60 hospitalized pediatric patients with malnutrition were enrolled in the study. The median age was 1.38 (0.67, 4.71) years. The majority of patients were under 10 years old. Thirty-nine (65%) patients were male. The first three common principal diagnoses were pneumonia (35%), cholestasis (18.3%), and sepsis (11.7%). Fifty-six (93.3%) patients had a medium risk of malnutrition according to the STRONGkids nutritional screening tool, and the remaining patients had a high risk; however, only twenty-nine (48.3%) patients suffered from malnutrition according to the WHO classification. The median time to achieve target energy and protein intake was 257 (150, 430) hours.

Fourteen patients (23.3%) who achieved target energy and protein intake within 7 days were categorized into the early achievement group, whereas the remaining patients were classified into the late achievement group. The median time to achieve the target energy and protein intakes was 108.5 (41, 150) hours in the early group and 394 (257, 798) hours in the late group. Among the 46 patients in the late achievement group, 19 patients did not reach the target energy and protein intakes during hospitalization. There was no statistically significant difference in age, sex, principal diagnosis, admission ward, signs of nutrient deficiency, fluid restriction, feeding intolerance, and receiving medication or surgery between the groups (Table 2). Additionally, there was no statistically significant difference in underlying diseases between the groups (*p* = 0.55).

The median LOS of patients in the early achievement group was significantly shorter than that of patients in the late achievement group. No significant difference in improvement in nutritional status or mortality was observed between the groups. Other demographic data of the patients are presented in Table 2.

**Fig. 2:**
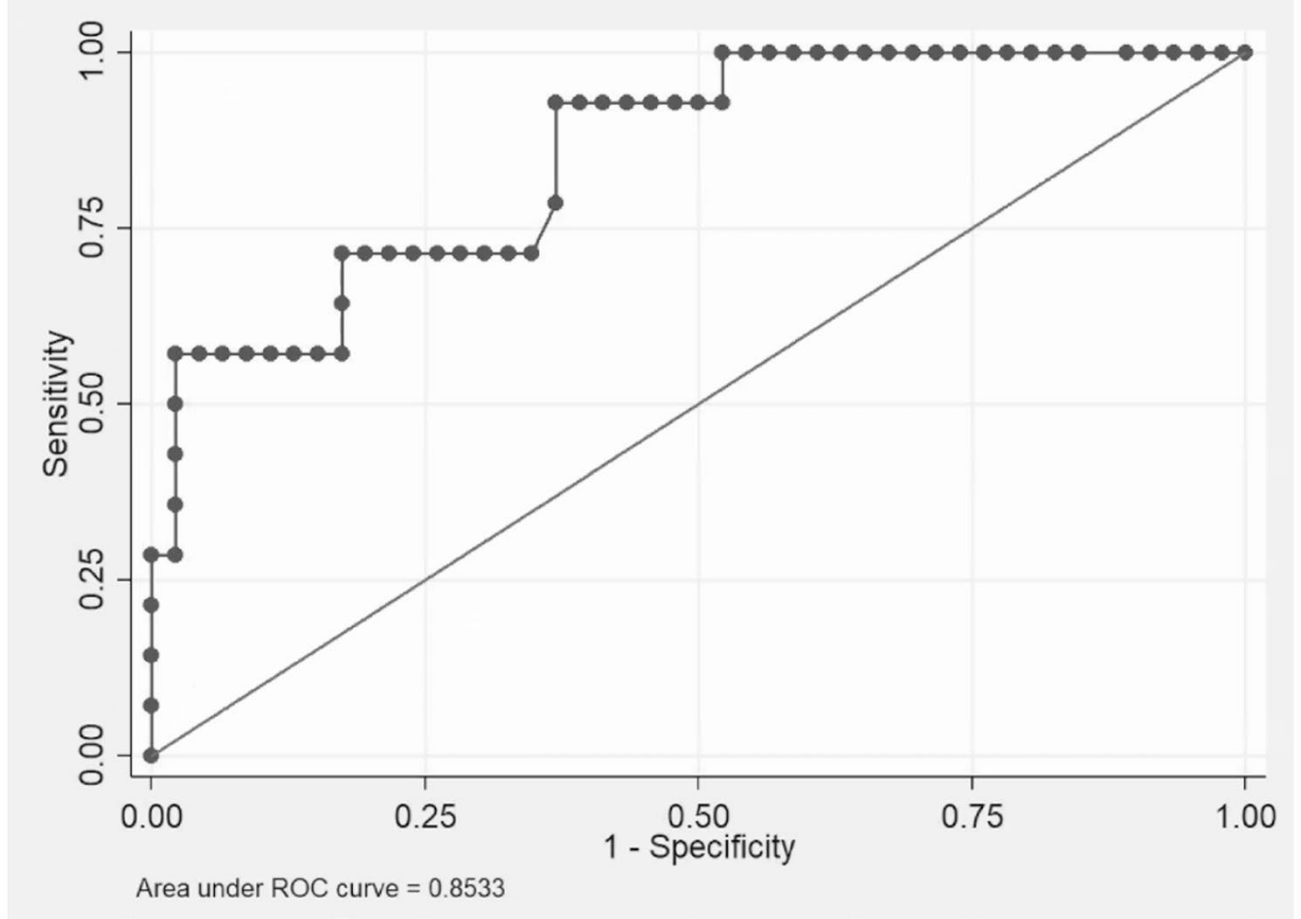
Logistic regression model of factors associated with the early achievement of target nutrient intakes. Logistic regression model of factors associated with early achievement of target energy and protein intake, including only statistically significant factors from the multivariable model: pre-existing nutritional intervention and weight-for-length/height z-score. The model demonstrated excellent discrimination, with an area under the receiver operating characteristic curve (auROC) of 0.853 and an Akaike’s information criterion (AIC) of 49.12. These factors contributed to the most appropriate model, achieving the highest auROC and the lowest AIC

According to the aforementioned findings, only four indicators demonstrated statistically significant differences between groups, namely item 3–3 of STRONGkids (*p* = 0.035), malnutrition (*p* = 0.005), weight-for-age z-score (*p* = 0.001) and weight-for-length/height z-score (*p* = 0.008) on the first day of admission. To determine the probable factors associated with early achievement of target energy and protein intake, those components were analyzed with a multivariate logistic regression model. The crude odds ratios (ORs) and adjusted ORs, along with their 95% confidence intervals (CIs), are presented in Table 3. However, the most accurate factors, which illustrated the least AIC and the most auROC in the curve, could be obtained by using only item 3–3 of STRONGkids and the weight-for-length/height z-score on the first day of admission. The auROC was 0.853, which is considered excellent discrimination; moreover, the AIC was 49.12, as shown in Fig. 2.

**Table 3:** Factors associated with early achievement of target energy and protein intakes^†^

Factors	Univariable model		Multivariable model	
	Odds ratio (95%CI)	*p*-value	Adjusted odds ratio (95%CI)	*p*-value
STRONGkids item 3–3^‡^	0.20 (0.04–0.98)	0.048*	0.03 (0–0.37.37)	0.006*
Weight-for-age z-score	2.11 (1.27–3.51)	0.004*		
Weight-for-length/height z-score	1.69 (1.16–2.46)	0.007*	2.39 (1.44–3.97)	0.001*
Malnutrition	0.12 (0.02–0.59)	0.009*		

* Significant differences between groups (*p* < 0.05)

^†^ Early achievement was defined as reaching the target within 7 days of admission,

^‡^ STRONGkids, Screening Tool for Risk on Nutritional Status and Growth:

(1) Subjective clinical assessment (0 or 1 point), (2) High-risk disease (0 or 2 points), (3) Nutritional intake and losses-reduced intake in the past few days, diarrhea, vomiting, or pre-existing nutritional intervention (0 or 1 point), and (4) Weight loss or poor weight gain (0 or 1 point). The total score ranges from 0–5, classified as low (0), moderate (1–3), and high (4–5) risk of malnutrition

### Evaluation of time consumed for nutritional management, satisfaction, and comfort by pediatric residents

**Fig. 3:**
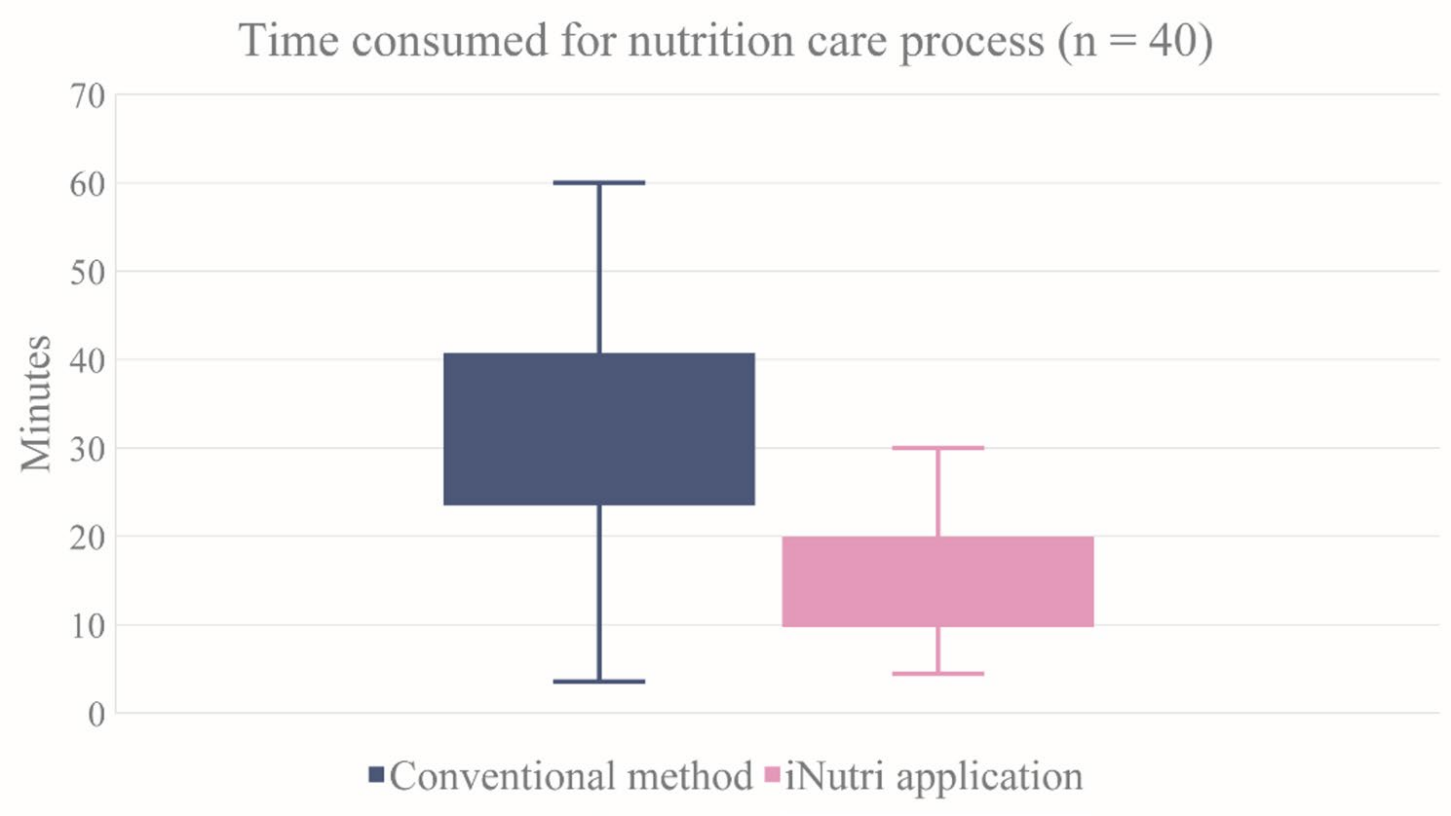
Time consumed for the nutritional care process. Time consumed for the nutritional care process was evaluated by 40 pediatric residents. The box plot illustrates the data about time consumption during the nutritional care process categorized by method. The data are expressed as medians (Q1, Q3). The blue color represents the conventional method, with 31.67 (22.5, 41) minutes, whereas the pink color represents the iNutri application, with 15 (10, 19.5) minutes. *p*-value < 0.001, Wilcoxon signed-rank test

A total of 40 pediatric residents volunteered to participate in this study. The median time to provide a standard nutritional care process was 31.67 (22.5, 41) minutes, whereas the median time to perform that action while using the iNutri application was 15 (10, 19.5) minutes. A significant difference in the median time between the two methods was noted (*p* < 0.001) and is illustrated in Fig. 3.

The survey of satisfaction and comfort with the iNutri application from the residents for all assessed items is summarized in Table 4.

**Table 4:** Scores of the resident survey on satisfaction and comfort with the iNutri application

Items	5-point Likert (Median (Q1, Q3))
Ease of utilizing iNutri	4.4 (4.0, 5.0)
Coverage in details	4.2 (4.0, 4.5)
Confidence to do 24-hour dietary recall	4.1 (4.0, 4.5)
Self-assurance to initiate EN or PN	4.0 (3.6, 4.0)
Obtaining knowledge about medical formula	4.0 (3.9, 4.5)
Understanding about blenderized diet	3.7 (3.0, 4.0)
Comprehension in portion size	4.0 (4.0, 4.3)
Recognition in PN	3.7 (3.2, 4.0)
Mastery in nutrition care process	4.0 (4.0, 4.0)

*EN* enteral nutrition, *PN* parenteral nutrition

## Discussion

In this single-arm pilot study, 23% of patients with malnutrition receiving care with the iNutri application achieved target energy and protein intakes within 7 days. The median time to achieve target intake was 257 h. Our data suggested that patients without pre-existing nutritional intervention (question item 3–3 of STRONGkids) were more likely to achieve target intakes early, whereas patients with a lower weight-for-length/height z-score tended to experience late achievement. Furthermore, the median LOS of patients in the early achievement group was shorter than that of patients in the late achievement group. Overall, the iNutri application demonstrated excellent reliability for estimating carbohydrate, protein, fat, and energy intake from dietary recall (ICCs of 0.998, 0.994, 0.998, and 0.999, respectively), and was associated with a reduction in the duration of the nutritional care process. Resident feedback also indicated levels of high satisfaction and comfort with the application.

One of the problematic issues in providing nutrition care is the duration of the acquisition of target nutrients. A previous retrospective study in the U.S. revealed that the use of EN application promoted patients to obtain EN earlier from 38.8% to 62.5% and increased opportunities to reach the target energy [17]. Another study, which compared nutrition education through applications with standard tools for colorectal cancer patients in Thailand, reported that the application had the potential to promote patients’ appetite and improve nutritional parameters [18]. The results revealed that novel technologies had the same benefit in assisting patients in achieving target energy and protein intake faster. In the present study, using iNutri application alongside conventional nutritional management was associated with earlier achievement, but a direct causal relationship could not be determined due to variations in disease severity and the single-arm study design. While not a predefined variable for analysis, we observed that patients in the late achievement group often had more complex underlying conditions and multiple comorbidities, such as septic shock with multiorgan failure, DGAT1 mutation, and complex heart disease. In contrast, patients with early achievement typically present with less complicated conditions, such as simple infections, which may be reflective of our hospital’s role as a tertiary center.

Factors associated with early achievement of target nutrient intake have been reported in the literature. A previous prospective study in the Netherlands, which included 325 PICU patients, also revealed that patients who underwent surgery and high severity of illness were associated with underfeeding [5]. Another prospective study in a Malaysian PICU (*n* = 139) identified longer PICU stay, duration of EN interruption, presence of fluid restriction, and children who underwent surgery as risk factors for lower energy intake [6]. Adult ICU studies also reported that patients receiving vasopressors achieved EN goals later [17]. In our study, a better weight-for-length/height z-score on the first day of admission and the absence of pre-existing nutritional intervention were potentially associated with earlier achievement. These findings are novel in the context of a comprehensive mobile application, although we cannot definitively conclude that these factors are causal. They may reflect the benefit of incorporating a nutritional screening tool in the care process; however, we cannot definitively conclude that these factors are essential for early target achievement, as patients with better baseline nutritional status or no prior nutritional intervention may have been more receptive to EN. Further studies are needed to clarify their impact.

The LOS data were inconsistent. Some studies reported that body mass index, length (or height)-for-age z-score below the median − 2SD at admission [4] and lower energy intake [6] were associated with a longer LOS, whereas other studies did not demonstrate a relationship between a shorter LOS and nutritional intervention [5, 17]. In our study, we found that the LOS was significantly shorter in the early achievement group. These inconsistent results may vary depending on various factors, including malnourished status, disease severity, age group, and nutritional intervention.

The duration of the nutritional care process needs to be addressed as a concern. A preceding study in 30 Spanish patients revealed that the mobile nutritional application was capable of saving time for performing nutritional assessments of approximately 2–3 min per case [14]. Another study, which was conducted in Brazil, demonstrated that the use of a digital application helped nutritionists detect nutritional risk in patients 250 s faster [19]. A study in Switzerland also reported that introducing the food-record mobile application to 12 parents was practical and sufficient information was obtained [24]. As a result, it was evident that the application had a benefit in this process. The present study suggested that utilizing the iNutri application is associated with optimizing the time required for nutrition care processes among pediatric residents, with an observed reduction of approximately 16 min per patient.

In fact, the number of mobile nutritional applications for children is relatively small, and their functions are limited. One study demonstrated that an application could help parents record food intake more accurately and provide sufficient data [24]. Another artificial intelligence-based application was designed to generate meal plans that met target nutrient requirements [25]. Other applications have focused primarily on eating habits, self-monitoring, or academic purposes [20–23]. However, to the best of our knowledge, our study introduces the first comprehensive mobile nutritional application for pediatric patients. It covers all aspects of the nutritional care process, including anthropometric measurements, nutritional assessments, 24-hour dietary recall, determination of target energy and protein intakes, and monitoring processes. Furthermore, this is the first study to use a comprehensive mobile nutritional application to provide nutritional care to pediatric inpatients with malnutrition. Additionally, this study observed factors potentially associated with achieving individualized target energy and protein intake by integrating clinical findings, nutritional screening tool, and nutritional status. Providing nutrition care is challenging and time-consuming for pediatric residents; this application may help streamline these processes and potentially support more efficient nutritional care. Moreover, determining target energy and protein intake is both crucial and challenging in clinical practice. Achieving these targets may be associated with a shorter LOS for patients. There are several strengths to the present study. Firstly, we developed the first comprehensive mobile nutritional application, which provide accurate data on nutritional assessment, 24-hour dietary recall, and suggested target nutrient intake. Secondly, it was associated with streamlining the time required for the nutritional care process for pediatric residents, with generally favorable performance feedback. Thirdly, we observed factors potentially associated with the early achievement of target energy and protein intake. These findings may indicate that clinicians should pay closer attention to patients with low weight-for-length/height or those who have previously received nutritional intervention. Finally, our study was conducted in a prospective manner, with a small sample size but larger than that of some previous studies [14, 24]. Despite these strengths, a limitation of the study was its single-arm design. We could not definitively conclude that the iNutri application directly accelerated the achievement of target nutrient intakes, as this study did not include a concurrent comparison with the conventional method. The small sample size further restricts our ability to draw definitive conclusions regarding the application’s clinical efficacy and the factors influencing early achievement of target intake. This study was primarily a pilot project designed to evaluate the feasibility and initial impact of the iNutri application, with the goal of informing the design and required sample size for a future, larger-scale, randomized controlled trial. A formal, predefined disease severity score was also not included in our analysis. Although we observed differences in the complexity of underlying conditions between the early and late achievement groups, a validated measure would be necessary for a definitive conclusion on its impact on the time to reach target nutrient intakes. While the application demonstrated good satisfaction scores, the internal consistency of the questionnaire was not assessed, and we acknowledge this as a limitation of our findings. Applying standardized measures of questionnaire usability in future studies could enhance and confirm its user-friendliness. Additionally, as a single-center study, our findings may not be fully generalizable to other institutions with different patient populations, workflows, or resources. Further large-scale studies are warranted to provide more robust evidence and identify factors influencing the early achievement of target energy and protein intake. Moreover, the young median age of our study population (1.38 years) relative to the broad inclusion criteria (1 month to 18 years) is a limitation. Although we aimed to recruit patients across a broader age range, older children in our setting tended to have a lower risk of malnutrition and were consequently underrepresented in our study. A significant methodological limitation pertains to the reliability testing of the 24-hour dietary recall. The analysis was subject to confounding between the rater’s professional expertise (dietitians for the paper-based method versus pediatricians for the app-based method) and the assessment method itself. This potential rater effect could influence the interpretation of the ICC estimates. Since a sensitivity analysis using standardized raters was not feasible, we explicitly acknowledge this limitation. However, we clarify that the high ICC values observed primarily serve to validate the iNutri application’s core calculation system and database, demonstrating its ability to reliably reproduce nutritional outcomes derived from the conventional, dietitian-led standard. Nonetheless, future validation studies must fully control for the rater variable by utilizing the same standardized, trained personnel for both methods to precisely quantify the inter-rater and inter-method agreement.

## Conclusions

The present study suggests the potential for the iNutri application to have positive effects on clinical nutritional care. The time required for nutritional assessment was reduced, which was associated with the potential to optimize the nutritional healthcare system.

## Supplementary Information

Supplementary Material 1.

Supplementary Material 2.

## Data Availability

Requests for deidentified data from individual participants are available through the corresponding author together with ethically approved proposals.

## Abbreviations

auROC: Area under receiver operating characteristic curve
AIC: Akaike’s information criterion
ARIETI: Aplicativo móvel de Representação da Ingestão alimEntar de pacienTes Internados
API: Application Programming Interface
EN: Enteral nutrition
INMUCAL: Institute of Nutrition, Mahidol University CALculation
ICC: Intraclass correlation coefficient
IRB: Institutional Review Board
KCMH: King Chulalongkorn Memorial Hospital
LOS: lengths of stay
NEPSA: Nutrition Educational Prototype based on Smartphone Web Applications
PN: Parenteral nutrition
PICU: Pediatric intensive care unit
STRONGkids: Screening Tool for Risk on Nutritional Status and Growth
SD: Standard deviation
WHO: World Health Organization
