# Supplementary material for: A comprehensive mobile nutritional application is associated with improved time efficiency and user experience in managing hospitalized children with malnutrition
Source: BMC Pediatr. 2025 Dec 16;26:57. doi: 10.1186/s12887-025-06423-9 (PMC12829223; doi:10.1186/s12887-025-06423-9)
Supplement: Supplementary file 1 — Supplementary Material 1. [file 12887_2025_6423_MOESM1_ESM.pdf]

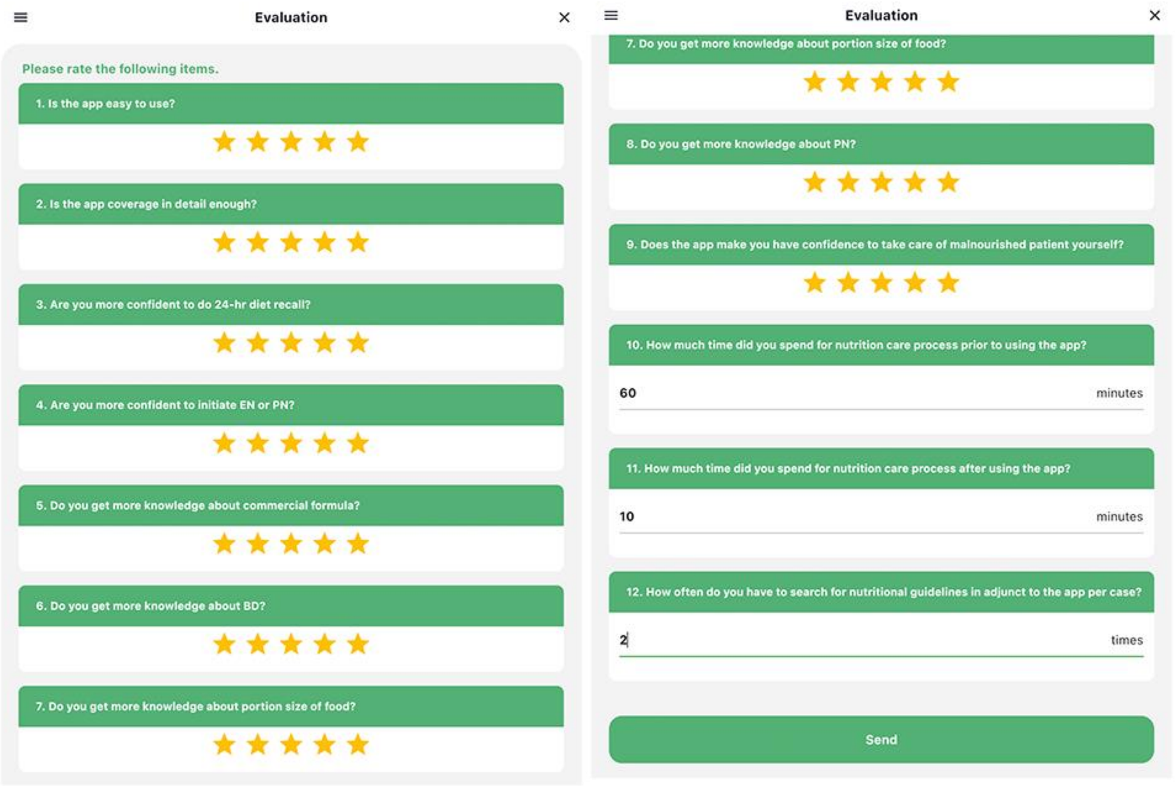

Supplemental Fig. 1 Evaluation survey of iNutri application usability and impact.

The structured satisfaction survey used to assess user feedback on the iNutri application was embedded within the app and contained 12 items. Items 1-9 were rated on a 5-point Likert scale, with higher scores indicating greater satisfaction, confidence, and knowledge. These questions evaluate the application's ease of use, content detail, and impact on the user's confidence in performing nutritional care processes, including dietary recall, initiating enteral/parenteral nutrition, and understanding nutritional concepts such as commercial formulas and portion sizes. Items 10-12 collected quantitative data on the time spent on nutritional care before and after using the app, as well as the frequency of needing to consult external nutritional guidelines.
