# Supplementary material for: A comprehensive mobile nutritional application is associated with improved time efficiency and user experience in managing hospitalized children with malnutrition
Source: BMC Pediatr. 2025 Dec 16;26:57. doi: 10.1186/s12887-025-06423-9 (PMC12829223; doi:10.1186/s12887-025-06423-9)
Supplement: Supplementary file 2 — Supplementary Material 2. [file 12887_2025_6423_MOESM2_ESM.pdf]

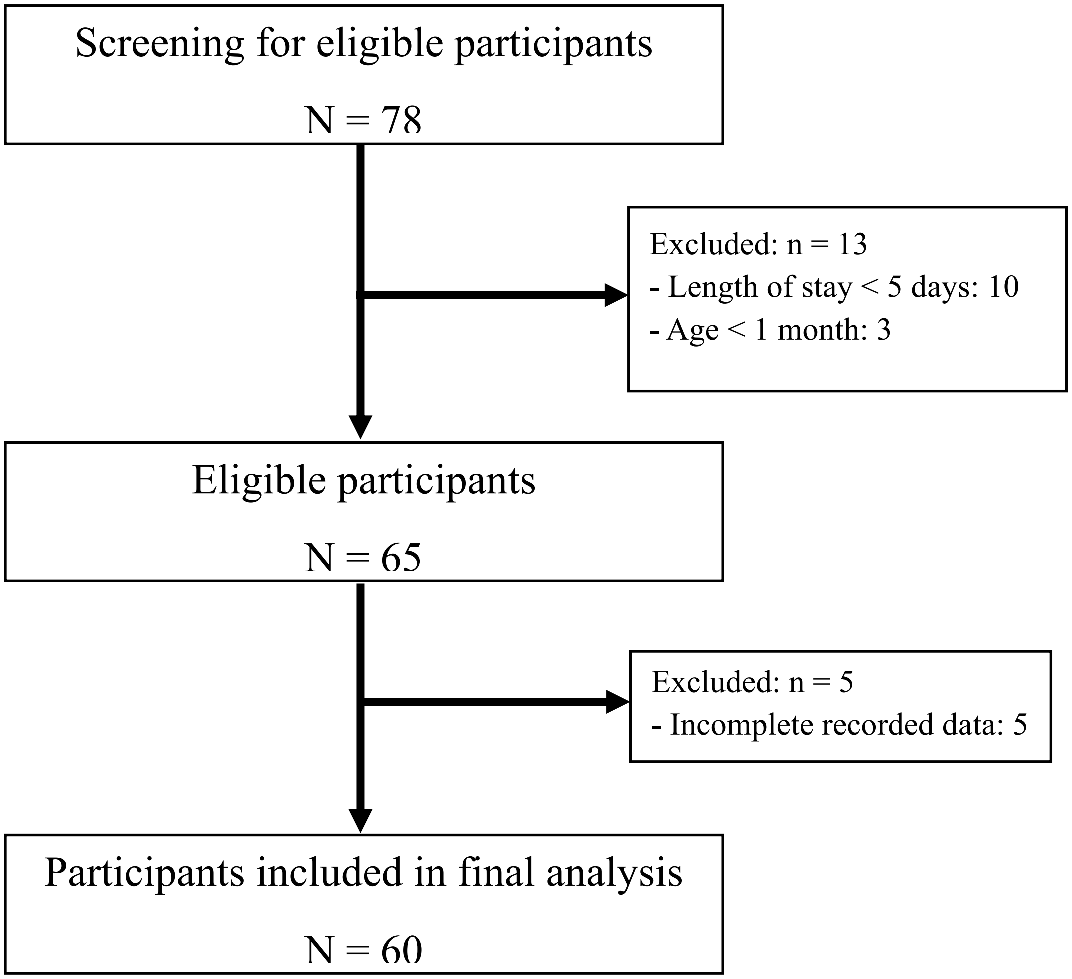

Supplemental Fig. 2 Flowchart of patient screening and enrollment.

The flowchart details the patient screening and enrollment process for this study. It provides the number of children initially screened, those who met the inclusion criteria, and the final number of patients included in the study.
